# Flexible parametric modelling of cause-specific hazards to estimate cumulative incidence functions

**DOI:** 10.1186/1471-2288-13-13

**Published:** 2013-02-06

**Authors:** Sally R Hinchliffe, Paul C Lambert

**Affiliations:** 1 Department of Health Sciences, Centre for Biostatistics and Genetic Epidemiology, University of Leicester, Leicester, UK; 2 Department of Medical Epidemiology and Biostatistics, Karolinska Institutet, Stockholm, Sweden; 3 Department of Health Sciences, Biostatistics Group, University of Leicester, Leicester, UK

**Keywords:** Competing risks, Flexible parametric model, Cause-specific hazards

## Abstract

**Background:**

Competing risks are a common occurrence in survival analysis. They arise when a patient is at risk of more than one mutually exclusive event, such as death from different causes, and the occurrence of one of these may prevent any other event from ever happening.

**Methods:**

There are two main approaches to modelling competing risks: the first is to model the cause-specific hazards and transform these to the cumulative incidence function; the second is to model directly on a transformation of the cumulative incidence function. We focus on the first approach in this paper. This paper advocates the use of the flexible parametric survival model in this competing risk framework.

**Results:**

An illustrative example on the survival of breast cancer patients has shown that the flexible parametric proportional hazards model has almost perfect agreement with the Cox proportional hazards model. However, the large epidemiological data set used here shows clear evidence of non-proportional hazards. The flexible parametric model is able to adequately account for these through the incorporation of time-dependent effects.

**Conclusion:**

A key advantage of using this approach is that smooth estimates of both the cause-specific hazard rates and the cumulative incidence functions can be obtained. It is also relatively easy to incorporate time-dependent effects which are commonly seen in epidemiological studies.

## Background

In epidemiological studies two main measures of interest are the risk of an event occurring (probability) and the rate at which it occurs (hazard) [1]. Patients will often be at risk from more than one mutually exclusive event and the occurrence of one of these may alter or prevent the probability of any other event occurring [2]. In this paper we focus on situations where the events are deaths from different causes and so it follows that any event will prevent the others from occurring. In this competing risks scenario, the cause-specific hazard will give the cause-specific mortality rate and the cumulative incidence function will give the proportion of patients at any one time that have died from a particular cause [3].

There are two main approaches to modelling competing risks [4]. The first is to model the cause-specific hazards and transform these to obtain the cumulative incidence function. The second is to model the cumulative incidence function directly [5]. We advocate the first approach as both the cause-specific hazards and the cumulative incidence function can provide a better understanding of risk factors and their effect on the population as a whole [1]. Cause--specific hazards can inform us about the impact of risk factors on rates of disease or mortality, while the cumulative incidence functions provide an absolute measure with which to base prognosis and clinical decisions on [6].

Competing risks analyses are being increasingly carried out in epidemiological studies. However, the methodology applied varies and is not always optimal. Often, separate analyses will be carried out for each competing event and only the cause-specific hazard ratios will be reported for each [7-9]. This method is not wrong if the researchers are only interested in the rate of disease or mortality. However, without estimating an absolute measure such as the cumulative incidence function, it is difficult to communicate these results in terms of the impact that risk factors have at a population level. In comparison, other researchers choose to model on the cumulative incidence scale using the Fine and Gray method and, therefore, provide no information on the cause-specific hazards [10,11].

In many research papers, the model used to estimate the cause-specific hazards will be different from the model used to estimate the cumulative incidence functions. For example, the cause-specific hazard ratios are reported from a Cox proportional hazards regression model but the cumulative incidence functions are estimated non-parametrically and separately for different subgroups of patient [12-14]. Whilst non-parametric approaches are good for describing the data, there are many advantages for the use of modelling techniques in observational studies when there are a number of covariates that need to be adjusted for.

Many regression models used to estimate cumulative incidence functions will assume proportional hazards. In large epidemiological studies the assumption of proportional hazards is often unreasonable. Therefore, a model that can easily incorporate time-dependent effects is desirable.

In summary, we would like to be able to model competing risks scenarios using the approach that estimates both the cause-specific hazards and the cumulative incidence functions as we believe both to be useful measures. We would like to obtain smooth estimates for both of these measures rather than considering a step function. Finally, we want to be able to incorporate time-dependent effects for one or all of the competing events. Whilst the majority of the above can be addressed within a Cox modelling framework, we feel that parametric models have the advantage of directly estimating cause-specific hazard rates in the model as well as handling non-proportional hazards with ease. For these reasons, we advocate the use of the flexible parametric survival model to obtain both the cause-specific hazards and the cumulative incidence function in a competing risks framework.

## Methods

### Competing risks

If we assume that a patient is at risk from *K* different causes, the cause-specific hazard for the *k*^*th*^ cause, *h*_*k*_(*t*) is the rate of failure at time *t* given that no failure from cause *k* or any of the *K*-*1* other causes has occurred [3]. When the competing events are death from different causes these can be thought of as mortality rates. The cause-specific hazard can be written as

(1) $$h_{k}\left(t\right)=\lim_{\mathrm{\Delta t}↓0}\frac{P\left(t\leq T<t+\mathrm{\Delta t},K=k|T\geq t\right)}{\mathrm{\Delta t}}$$

Assuming proportional hazards, the cause-specific hazard rate for cause *k* for a patient with covariates x_*k*_ can be calculated using the equation

(2) $$h_{k}\left(t|x\right)=h_{k,0}\left(t\right)\exp\left(\beta_{k}x_{k}\right)$$

where *h*_*k*,*0*_(*t*) is the baseline cause-specific hazard for cause *k* and *β*_*k*_ are the covariate effects (log hazard ratios).

Once the cause-specific hazard has been estimated, many researchers will transform to obtain a survival function, *S*_*k*_(*t*), through the following transformation

(3) $$S_{k}\left(t\right)=\exp\left(-\int_{0}^{t}h_{k}\left(u\right)\mathrm{du}\right)$$

Under the assumption that the competing events are independent (conditional on covariates), the complement of the cause-specific survival function can be interpreted as the probability of dying from cause *k* in a hypothetical world where it is not possible to die from anything else [15]. In many situations the assumption of independence will not be reasonable in which case any estimates obtained through Equation (3) are not interpretable as probabilities. Even under the strong assumption of independence, these estimates of cause-specific survival are of little use to patients making decisions in the real world where death from other causes play a big role. Therefore, a better approach may be to acknowledge that patients may die from something else other than their cancer.

The cumulative incidence function, *C*_*k*_(*t*), gives the proportion of patients at time *t* who have died from cause *k* accounting for the fact that patients can die from other causes.

(4) $$C_{k}\left(t|x\right)=\int_{0}^{t}h_{k}\left(u|x\right)\exp\left(\int_{0}^{u}\Sigma_{k=1}^{K}h_{k}\left(v|x\right)\mathrm{dv}\right)\mathrm{du}$$

The cumulative incidence function is not only a function of the cause-specific hazard for the event of interest but also incorporates the cause-specific hazards for the competing events [1]. Previous research has mainly focussed on the use of the Cox model or non-parametric estimates in a competing risks framework [16,17]. Here, we advocate the use of the flexible parametric model.

### Flexible parametric model

We could apply Equation (4) to any standard parametric model; however, there are very few real world examples where all of the competing events can be adequately captured using a Weibull or exponential model for example. The flexible parametric survival model was first proposed by Royston and Parmar [18] for use with censored survival data. They proposed a range of models on different scales. We concentrate on models on the log cumulative hazard scale where the idea was to extend the Weibull model, which is a parametric proportional hazards model often criticised for the lack of flexibility in the shape of the baseline hazard function. Using a Weibull distribution the survival function can be written as

(5) $$S\left(t\right)=\exp\left(-{\mathrm{\lambda t}}^{\gamma }\right)$$

Transforming this to the log cumulative hazard scale gives

(6) $$\ln\;H\left(t\right)=\ln\left(\lambda \right)+\gamma \ln\left(t\right)$$

This is now a linear function of log-time. However, rather than assuming linearity with ln(*t*) the flexible parametric model uses restricted cubic splines for ln(*t*) [19]. The log cumulative hazard function is used as opposed to the hazard function as the “end artefacts” in the fitted spline functions at the extremes of the time scale are more severe for the hazard function. Furthermore, implementing on the log time scale means that the fitted function is typically gently curved or nearly linear, and is usually very smooth [18]. Finally, modelling on this scale means it is easy to transform to the survival and hazard functions [20].

Regression splines are piecewise polynomial functions that are forced to join at predefined points on the x-axis. These joining points are known as knots. In order to obtain a smooth function the regression splines are also forced to have continuous first and second derivatives. For restricted cubic splines a further restriction forces the splines to be linear beyond the boundary knots.

A restricted cubic spline function, *s*(ln(t)|**γ**, **n**_**0**_), with N knots, a vector of knots **n**_**0**_ and parameters γ_0,⋯,_γ_N - 1_ can be written as

(7) $$s\left(\ln\left(t\right)|\gamma ,n_{0}\right)=\gamma_{0}{+\gamma }_{1}z_{1}+\cdots +\gamma_{N-1}z_{N-1}$$

The derived variables z_1_ ⋯ z_N - 1_ are calculated as follows

(8) $$z_{1}=\ln\left(t\right)$$

(9) $$\begin{array}{l} z_{j}={\left(\ln\left(t\right)-n_{j}\right)}_{+}^{3}-\emptyset_{j}{\left(\ln\left(t\right)-n_{1}\right)}_{+}^{3}\\ \;-\left(1-\emptyset_{j}\right){\left(\ln\left(t\right)-n_{N}\right)}_{+}^{3},\;j=2,\cdots N-1 \end{array}$$

where

(10) $$\emptyset_{j}\frac{n_{N}-n_{j}}{n_{N}-n_{1}}$$

and (u)_+_ = u if u > 0 and 0 if u ≤ 0. Thus, a model with N knots for the baseline log cumulative hazard uses N-1 degrees of freedom.

The baseline log cumulative hazard in a proportional hazards model incorporates the restricted cubic spline function of *s*(ln(*t*)|**γ**, **n**_**0**_), with knot locations **n**_**0**_, and covariates **x** and can be written as

(11) $$\ln\left[H\left(t|x\right)\right]=s\left(\ln\left(t\right)|\gamma ,n_{0}\right)+x\beta$$

Covariate effects can be interpreted as log hazard ratios here under the assumption of proportional hazards. The survival and hazard functions can be obtained through a transformation of the model parameters

(12) $$S\left(t|x\right)=\exp\left(-\exp\left(\ln\left[H\left(t|x\right)\right]\right)\right)$$

(13) $$h\left(t|x\right)=\frac{\mathrm{ds}\left(\ln\left(t\right)|\gamma ,n_{0}\right)}{\mathrm{dt}}\exp\left(\ln\left[H\left(t|x\right)\right]\right)$$

One of the main advantages of the flexible parametric approach is the ease with which time-dependent effects can be fit [21]. Time-dependent effects can be incorporated into the model by forming interactions between covariates and restricted cubic splines for ln(t) with knots, **n**_**j**_, at centiles of the event times. If there are D time-dependent effects, then we can extend Equation (11) as follows:

(14) $$\ln\left[H\left(t|x\right)\right]=s\left(\ln\left(t\right)|\gamma ,n_{0}\right)+\mathrm{x\beta}+\Sigma_{j=1}^{D}s\left(\ln\left(t\right)|\delta_{j},n_{j}\right)x_{j}$$

The number of spline variables for a particular time-dependent effect will depend on the number of knots, **n**_**j**_[15].

As shown in Equation (4), the cumulative incidence is a function of the cause-specific hazard functions. The cause-specific hazard function can be obtained from the flexible parametric model through Equation (13) by only considering one cause of death at a time and censoring competing events. Alternatively, we can stack the data and fit one model for all *K* causes simultaneously. This approach is described in further detail later in the paper.

The integral in Equation (4) can be obtained numerically. The integration is performed using similar methods to those proposed by Carstensen [22] and Lambert et al. [15]. The formulae for these methods are given in Appendix 1. It is possible to construct confidence intervals for the cumulative incidence function under the Cox model [23]. However, this is by no means a trivial task [23,24]. An advantage of our approach is that confidence intervals can be obtained using the delta method as the baseline hazards are estimated as part of the model (see Appendix 1).

Two user-friendly commands have been written in Stata that implement the methodology described in this paper. The command stpm2 will fit a flexible parametric survival model [21] and the command stpm2cif can be used to obtain the cumulative incidence functions through post-estimation [25]. Example code for these commands can be found in Appendix 2.

### Relative measures

Once the cause-specific hazards and the cumulative incidence function have been estimated it is possible to obtain other useful measures through some simple manipulation of the estimates. The relative contribution to the total mortality can be derived as:

(15) $$\frac{C_{k}\left(t\right)}{\Sigma_{k=1}^{K}C_{k}\left(t\right)}$$

This can be interpreted as the probability of having died from cause *k* given that a death has occurred **by** time *t*.

The relative contribution to the overall hazard can be derived as:

(16) $$\frac{h_{k}\left(t\right)}{\Sigma_{k=1}^{K}h_{k}\left(t\right)}$$

This can be interpreted as the probability of having died from cause *k* given that a death has occurred **at** time *t*.

### Illustrative example

One research area that is increasingly making use of competing risks methodology is population based cancer studies. Here we use data obtained from the SEER public use dataset [26] on survival of breast cancer patients. The patients analysed were all white females aged between 18 and 103 and were diagnosed between the years 1996 and 2005. Patients that were diagnosed at death or autopsy (n = 509) or had an unknown cause of death (n = 546) were excluded from the analyses. Only patients with a first primary malignant indicator were included (n = 18,433 excluded). If the stage of breast cancer was unknown then the patient was also excluded (n = 991). This left a total of 38,544 patients to be analysed.

Cause of death was categorised into breast cancer, other cancer, diseases of the heart and other causes. Age at diagnosis was categorised into the groups 18–59, 60–69, 70–79 and 80+. Staging of the cancer was classified as localised, regional or distant. Diagnosis of breast cancer was considered as the time origin and follow-up was restricted to 10 years. Table 1 gives the number of patients within each age group and stage of cancer.

**Table 1:** **Number** (%) **of patients in each age group and stage of breast cancer at diagnosis**

**Age group**	**Localised**	**Regional**	**Distant**	**Total**
18-59	10,712 (55.6)	7,467 (38.8)	1,084 (5.6)	19,263 (100)
60-69	5,249 (64.3)	2,414 (29.6)	490 (6.1)	8,153 (100)
70-79	4,884 (68.1)	1,884 (26.2)	411 (5.7)	7,179 (100)
80+	2,645 (67)	983 (24.9)	321 (8.1)	3, 949 (100)
Total	23,490	12,748	2,306	38,544

It is possible to fit 4 separate models, one for each cause, to obtain 4 cause-specific hazards. However, to allow for potential shared covariate effects over two or more causes we can fit one model for all 4 causes simultaneously. In order to do this the data needs to be stacked so that each individual patient has 4 rows of data, one for each of the 4 causes [16]. Table 2 illustrates how the SEER breast cancer data should look once it has been stacked. Each patient has the opportunity to fail from one of four causes. Patient 1 is at risk from all four causes for 10 years but does not experience any of them and so is censored. Patient 2 is at risk from all four causes for 6.5 years but then dies from heart disease and so is no longer at risk from any of the four causes.

**Table 2:** Expanding the data set

**ID**	**Age**	**Time**	**Cause**	**Status**
1	50	10	Breast Cancer	0
1	50	10	Other Cancer	0
1	50	10	Heart Disease	0
1	50	10	Other Causes	0
2	70	6.5	Breast Cancer	0
2	70	6.5	Other Cancer	0
2	70	6.5	Heart Disease	1
2	70	6.5	Other Causes	0

## Results and discussion

### Proportional hazards models

Both a Cox-proportional hazards model and a flexible parametric proportional hazards model were fitted in order to make a comparison of the two models in terms of both the cause-specific hazard ratios and the cumulative incidence function. The Cox proportional hazards model does not directly estimate the baseline hazard, h_k,0_(t), therefore, when obtaining the cumulative incidence functions the Breslow method for the cumulative baseline hazard needs to be substituted into Equation (4). However, if the cause-specific hazard rates were required then the baseline hazards would need to be estimates through post-estimation using, for example, kernel smoothing [21]. For the flexible parametric model the baseline knots were positioned differently for each of the four causes. The knot locations were chosen by taking the first and last event times along with the 25^th^, 50^th^ and 75^th^ centiles of the event times for each of the four causes.

As shown in Table 2, the data has been stacked so that each patient now has four rows of data, one for each cause. If the effects of age and stage were believed to be the same for each of the four causes of death then the stacked data format would allow us to share the parameters across all four causes. However, in this example, the effects of both age group and stage at diagnosis are different for each cause. We could revert to fitting a separate model for each of the four causes of death but for demonstrative purposes we have instead fitted interaction terms between each cause and each of the two variables. Further details of this can be seen in the Stata code in Appendix 2.

Table 3 gives the hazard ratios from both the Cox proportional hazards model and the flexible parametric proportional hazards model. The hazard ratios and their confidence intervals are very similar for both models. It is well known that mortality rates increase with age at diagnosis and this is evident for all four causes of death in this case. The results also show that the rate of death for all four causes increases with severity of breast cancer staging.

**Table 3:** **Comparison of Cox proportional hazards model** (**Cox**) **and flexible parametric proportional hazards model** (**FPM**), **hazard ratios** (**95**% **confidence intervals**)

	**Breast cancer**		**Other cancer**	
	**Cox**	**FPM**	**Cox**	**FPM**
Ages 18-59	1.00	1.00	1.00	1.00
Ages 60-69	0.90 (0.83, 0.97)	0.90 (0.83, 0.98)	2.12 (1.52, 2.94)	2.12 (1.52, 2.95)
Ages 70-79	1.27 (1.17, 1.37)	1.27 (1.17, 1.37)	3.18 (2.31, 4.37)	3.19 (2.32, 4.38)
Ages 80+	2.08 (1.90, 2.28)	2.09 (1.91, 2.29)	6.59 (4.73, 9.17)	6.63 (4.76, 9.23)
Localised	1.00	1.00	1.00	1.00
Regional	4.15 (3.85, 4.47)	4.15 (3.85, 4.47)	2.15 (1.61, 2.88)	2.16 (1.61, 2.88)
Distant	33.68 (31.08, 36.50)	33.84 (31.23, 36.67)	25.58 (19.18, 34.12)	25.82 (19.36, 34.44)
	**Heart disease**		**Other causes**	
	**Cox**	**FPM**	**Cox**	**FPM**
Ages 18-59	1.00	1.00	1.00	1.00
Ages 60-69	4.76 (3.62, 6.24)	4.76 (3.62, 6.24)	3.46 (2.89, 4.14)	3.46 (2.89, 4.14)
Ages 70-79	17.05 (13.42, 21.67)	17.07 (13.43, 21.69)	10.22 (8.73, 11.96)	10.22 (8.73, 11.96)
Ages 80+	70.57 (55.84, 89.17)	70.75 (55.99, 89.40)	31.54 (27.00, 36.84)	31.60 (27.07, 36.91)
Localised	1.00	1.00	1.00	1.00
Regional	1.42 (1.27, 1.60)	1.42 (1.27, 1.60)	1.11 (1.01, 1.26)	1.11 (1.02, 1.22)
Distant	2.44 (1.89, 3.14)	2.46 (1.91, 3.16)	2.08 (1.67, 2.58)	2.09 (1.68, 2.60)

**Figure 1:**
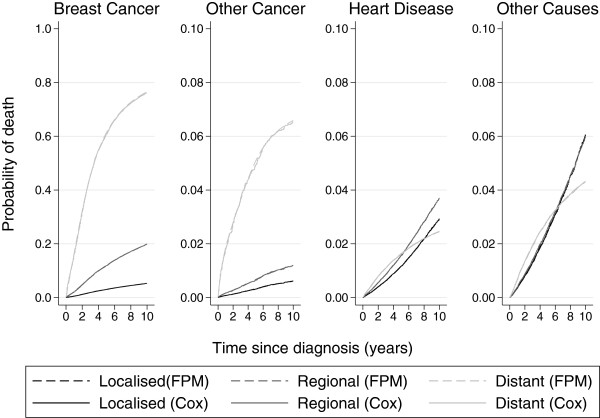
**Comparison of Cox proportional hazards model (Cox) and flexible parametric proportional hazards model (FPM) for ages 60–69.** Note: breast cancer is on a different scale.

Figure 1 shows the cumulative incidence functions for each of the four causes of death broken down by stage for patients aged 60–69. The estimates taken from the Cox model and the flexible parametric model are so similar that the two sets of curves overlay each other.

**Figure 2:**
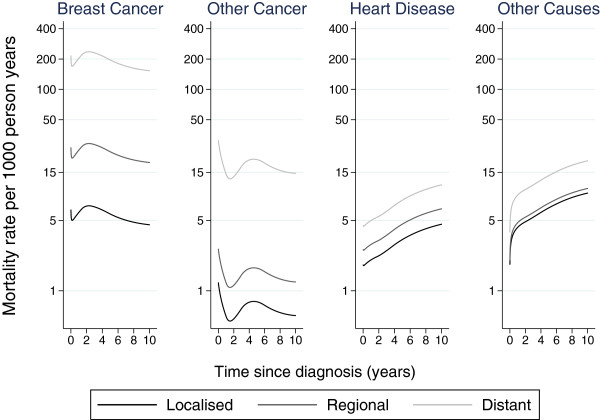
**Cause-specific hazard functions by breast cancer stage for ages 60–69 taken from the flexible parametric proportional hazards model.**

Figure 2 shows the cause-specific hazards from the flexible parametric proportional hazards model for ages 60–69 by stage at diagnosis. As follow-up time increases, the mortality rate for breast cancer decreases for all three stages. However, the mortality rate for heart disease and other causes increases with time.

Previous studies have shown a relationship between radiation therapy and cardiovascular mortality [27-29] and a similar relationship for chemotherapy [30]. The likelihood of receiving either radiotherapy or chemotherapy as a treatment for breast cancer increases with the severity of the staging. This could again explain the increased risk of death from heart disease with increasing severity of breast cancer staging [31].

Figure 2 illustrates how the proportional hazard assumption forces the log hazard functions for the three stages to be parallel to each other. We can relax this assumption by incorporating time-dependent effects in the model.

### Time-dependent models

For the remaining analyses we only considered a flexible parametric non-proportional hazards model. This model included time-dependent effects for age groups 60–69, 70–79 and 80+ for breast cancer and other causes and also for regional and distant stages for breast cancer, other cancer and other causes. These were selected using likelihood ratio tests (p-value < 0.05). All the time-dependent effects were fitted using 4 degrees of freedom and had the same knot locations as those used in the proportional hazards model.

**Figure 3:**
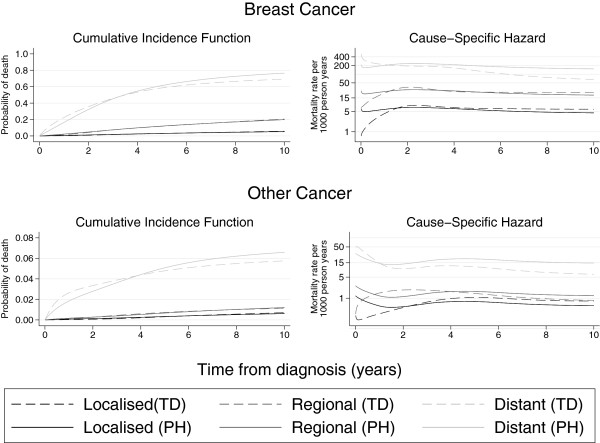
**Comparison of proportional hazards model (PH) and model incorporating time**-**dependent effects (TD) using the flexible parametric survival model for ages 60–69.** Note that the plots for breast cancer and other cancer are on different scales.

Figure 3 shows the cumulative incidence function and the cause-specific hazard function for both breast cancer and other causes of death. Separate curves are given for each of the three stages; localised, regional and distant. The figure compares estimates from the proportional and non-proportional flexible parametric models for those aged 60–69. It is evident from the cause-specific hazard function that incorporating time-dependent effects allows for more flexibility for the hazards over time and that the proportional hazards assumption is not reasonable. The differences between the proportional and non-proportional hazards models in terms of the cumulative incidence function are also visible. For example, reading from Figure 3 the probability of death from breast cancer for those aged 60–69 with distant stage cancer at 10 years post diagnosis is approximately 0.75 in the proportional hazards model but approximately 0.7 in the non-proportional hazards model - a difference of 0.05.

**Figure 4:**
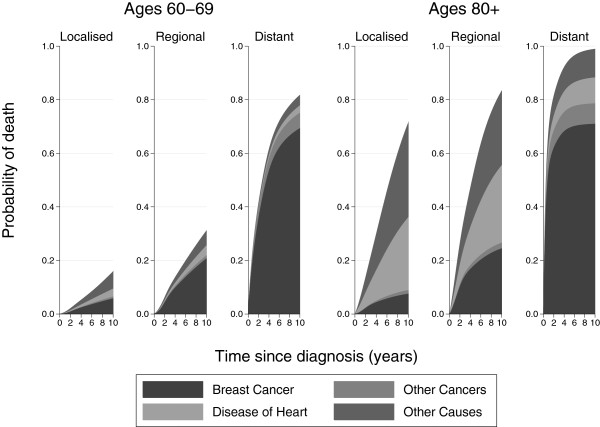
**Stacked cumulative incidence function plots by stage for ages 60–69 and 80 + .**

Figure 4 shows the cumulative incidence functions for each cause stacked on top of each other for the age groups 60 to 69 and 80+. This allows us to visualise the total probability of death and see how it is broken down by the different causes. If we concentrate on localised stage breast cancer, the total probability of death at 10 years for those aged 60–69 is 0.16 compared to 0.71 for those aged 80+. For those aged 60–69 with regional stage cancer, the most common cause of death is breast cancer. However, for those aged 80+ with regional stage cancer, deaths from heart disease and other causes are just as prominent as deaths from breast cancer.

### Relative measures

**Figure 5:**
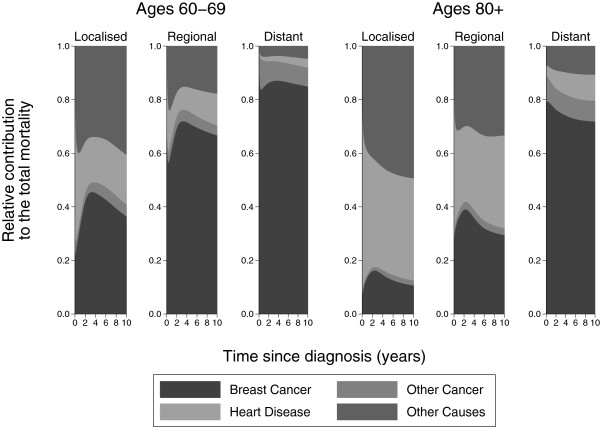
**Relative contribution to the total mortality by stage for ages 60–69 and 80 + .**

Figure 5 shows the contribution to the total mortality for ages 60–69 and 80+. There is a clear peak in the probability of dying from breast cancer in the localised and regional stage groups. Focussing on regional stage cancer, by 6 years after diagnosis from breast cancer, if a patient aged 60–69 has died then there is a probability of 0.7 that it was from breast cancer, 0.04 that it was from another cancer, 0.1 that it was from diseases of the heart and 0.16 that it was from other causes. If a patient aged 80+ has died by 6 years then the probability it was from breast cancer is 0.32, from another cancer is 0.03, from diseases of the heart is 0.32 and from other causes is 0.33.

**Figure 6:**
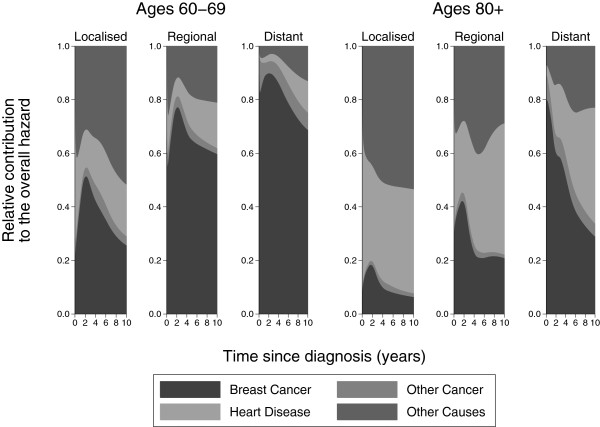
**Relative contribution to the overall hazard by stage for ages 60–69 and 80 + .**

Figure 6 shows the contribution to the overall hazard. Notice that there is a steeper decline in the proportion of breast cancer deaths compared to Figure 5 as we are now considering the instantaneous risk of death from each cause. If we focus on regional stage cancer if a patient aged 60–69 dies at 6 years then there is a probability of 0.63 that it was from breast cancer, 0.03 that it was from a another cancer, 0.14 that it was from diseases of the heart and 0.2 that it was from other causes. If a patient aged 80+ dies at 6 years then the probability it was from breast cancer is 0.21, from another cancer is 0.02, from diseases of the heart is 0.38 and the from other causes is 0.39.

### Confidence intervals

**Figure 7:**
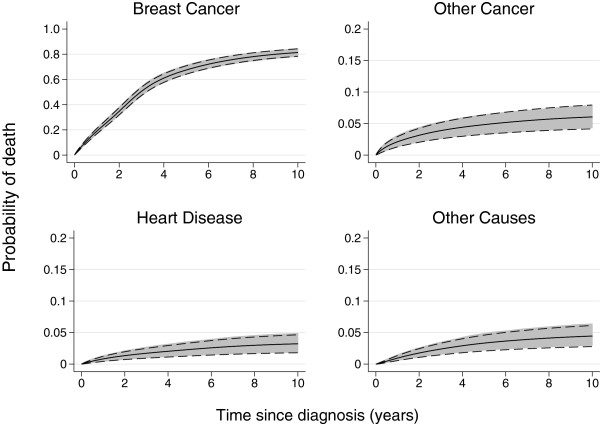
**Comparison of 95 per cent confidence intervals for the cumulative incidence function using the delta method (dashed lines) and bootstrapping (shaded area).** Note: breast cancer is on a different scale.

Figure 7 shows the estimated cumulative incidence functions and corresponding 95 per cent confidence intervals for breast cancer, other cancers, heart disease and other causes for those aged 60 to 69 with distant stage cancer. The confidence intervals were calculated using the delta method as described in the Appendix and also by using bootstrapping with 1000 replications. The bias-corrected method was used to calculate the percentile-based bootstrapped confidence intervals [32]. In order to speed up the bootstrap process, the estimations were carried out on a subset of the data where only patients in the age group 60–69 were considered. The figure clearly indicates that the two methods show agreement in both the upper and lower bounds of the confidence interval. The bootstrapped confidence intervals took a considerably longer amount of time to estimate than those obtained through the delta method (just over one hour for the bootstrapping as opposed to a couple of seconds for the delta method). Using bootstrapping on the full data set would take substantially longer.

### Sensitivity to number of knots

**Figure 8:**
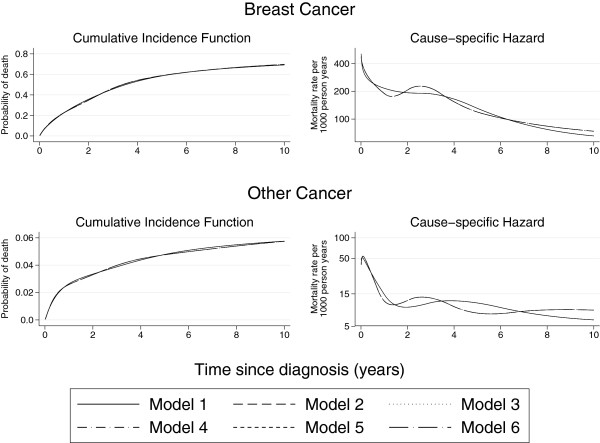
**Comparison of models with varying numbers of knots for distant stage, ages 60–69.** Note that, although there are 6 curves plotted on the graph, 5 curves are overlaying on the cause-specific hazard plots and only model 6 differs from the other models.

All the non-proportional hazard analyses in this paper were carried out using 4 degrees of freedom for both the baseline effects and the time-dependent effects. As a sensitivity analysis, four further models were fitted that compared the number and locations of the knots for the baseline effects and the time-dependent effects of age group and stage. Table 4 describes the models used in the sensitivity analysis. Model 1 refers to the non-proportional hazards model used throughout this paper. In terms of the AIC, model 1 is the best fitting model but in terms of the BIC, model 4 is the best fitting model. However, Figure 8 demonstrates that, with exception to model 6, the overall shape of the cause-specific hazard function is very much the same and the choice of model has little impact on the cumulative incidence function. Model 6 only considers 3 degrees of freedom for both the baseline effects and the time-dependent effects and so is most likely not able to fully capture the shapes of the underlying baseline hazards for the 4 causes.

**Table 4:** **Models with varying degrees of freedom for the baseline time**-**dependent effects**, ***df***_***b ***_**and the additional time**-**dependent effects**, ***df***_***t***_

	**Baseline*df***_***b***_	**Time**-**dependent*df***_***t***_	**AIC**	**BIC**
Model 1	4	4	**61841**.**19**	62459.84
Model 2	5	5	61945.39	62606.23
Model 3	5	3	61963.30	62483.53
Model 4	7	3	61947.53	**61783**.**53**
Model 5	7	4	61938.33	62585.10
Model 6	3	3	61962.75	62426.74

For 3 *df* knots are placed at centiles (0, 33, 67, 100), for 4 *df* at centiles (0, 25, 50, 75, 100), for 5 *df* at centiles (0, 20, 40, 60, 80, 100) and for 7 *df* at centiles (14, 29, 43, 57, 71, 86). These are placed on the distribution of uncensored event times.

## Conclusions

We have shown how to estimate both the cause-specific hazards and the cumulative incidence functions using a flexible parametric survival model. This approach provides smooth estimates of the cause-specific hazard and the cumulative incidence function, both of which we consider to be measures of interest. The flexible parametric model can easily incorporate time-dependent effects for one or more of the competing events. We have also illustrated two other useful measures that can be obtained with some simple manipulation of the cause-specific hazard and cumulative incidence estimates.

The flexible parametric proportional hazards model produces very similar estimates to the Cox proportional hazards model in terms of both the cause-specific hazard ratios and the cumulative incidence functions. A further alternative is to use a mixture model for competing risks data as proposed by Larson and Dinse [4,33]. However, this approach has two main disadvantages: it is time consuming and the estimated distribution will depend on the length of follow-up [34].

The confidence intervals obtained through the delta method have been shown to be very similar to those obtained through bootstrapping but have the added advantage of taking considerably less time to compute.

The assumption of proportional hazards is often unreasonable in epidemiological studies. It is important to understand the changing effect of a covariate over the time period rather than just assuming a constant hazard. For example, a treatment may have a large impact on mortality early on in the follow-up period but this effect could diminish as time goes on [35]. It is, therefore, important to consider methods such as those described in this paper, that can account for time-dependent effects. The flexible parametric model may be criticized as the number and location of the knots are subjective. However, the sensitivity analysis demonstrates that the knot location has very little impact in terms of the cumulative incidence function. Similar results have been reported elsewhere in relation to the sensitivity of the knots [15,18,20,36].

In this paper we have grouped age into four categories for simplicity whilst illustrating the method. However, it may be preferable to model age continuously using regression splines as has been done in previous papers [37,38].

The main advantages of the flexible parametric model are in large studies where time-dependent effects will often play a prominent role. In much smaller studies where there are fewer events there may not always be sufficient information to adequately estimate the underlying hazard using this model.

This paper describes modelling cause-specific hazards and using these to obtain the cumulative incidence function. Alternatively, the cumulative incidence function can be modelled directly using, for example, Fine and Grays subdistribution approach [5]. This may be useful when interest only lies in obtaining estimates of the cumulative incidence function for one of the competing events. However, if interest lies in visualising the overall probability broken down by specific events, such as those shown in Figure 2, then it should be noted that the direct regression approach does not have a boundary condition and so in some cases the overall probability may exceed one. We believe that the cause-specific approach, as described here, is advantageous for a full understanding of risk factors and real world implications.

Unlike measures of net survival, the cumulative incidence function allows us to present “real world” probabilities where a patient is not only at risk of dying from their cancer but also from any other cause of death. We can also estimate these “real world” probabilities using relative survival [15]. The advantage of the cause-specific approach is that we can examine more causes of death but this is at the expense of having to rely on cause of death information.

Finally, a user friendly program has been written in Stata to enable users to implement the methodology described in this paper. This command is called stpm2cif and is available from the Statistical Software Components (SSC) archive [25,39].

## Appendix 1–Details of the delta-method used to calculate confidence intervals

The integral in Equation (4) can be obtained numerically. Using similar methods to those proposed by Carstensen [22] and Lambert et al. [15] the integration is performed through the following steps:

1. The time scale is split into a large number, *m*, of small intervals.

2. The integrand of the cumulative incidence function, $\hat{f}\left(t_{m}|x_{0}\right)$, is predicted for a particular covariate vector, **x**_**0**_ at each of the *m* time intervals, *t*_*m*_.

3. The variance-covariance matrix for the integrand $\hat{f}\left(t_{m}|x_{0}\right)$, is obtained at each time interval using the delta method. The Stata command predictnl calculates the observation-specific derivatives for each parameter in the model. If we let *G* be the *m* × *p* matrix of observation-specific derivatives then the variance-covariance matrix can be estimated using the equation

$$\mathrm{Var}\left(\hat{f}\left(t_{m}\right)\right)=G\hat{V}G$$

where $\hat{V}$ is the estimated variance matrix for the model parameters.

4. The cumulative incidence function can then be calculated by summing the values of the integrand for the *m* time intervals. In order to do this, a triangular matrix *L* needs to be created. For example, for three intervals this looks like

$$C_{k}\left(t\right)=l\times \left[\begin{array}{rrr} 1 & 0 & 0\\ 1 & 1 & 0\\ 1 & 1 & 1 \end{array}\right]\;\left[\begin{array}{c} \hat{f}\left(t_{1}\right)\\ \hat{f}\left(t_{2}\right)\\ \hat{f}\left(t_{3}\right) \end{array}\right]\;=L\;\left[\begin{array}{c} \hat{f}\left(t_{1}\right)\\ \hat{f}\left(t_{2}\right)\\ \hat{f}\left(t_{3}\right) \end{array}\right]$$

where *l* is the interval length.

5. The variance-covariance matrix for the cumulative incidence function of the *k*^*th*^ cause is then calculated using

$$\mathrm{Var}\;(C_{k}\left(t\right)=\mathrm{LG}\hat{V}G^{\prime }L^{\prime }$$

## Appendix 2–Stata analysis code for flexible parametric model section of illustrative example. For more information see the Stata help file [38] or the Stata Journal article [30]

***Expand the data so that each patient has 4 rows – one for each cause of death***

expand 4

bysort id: gen cause = _n

***Generate indicator variables for each cause of death along with an overall indicator ***

gen breast = cause==1

gen cancer = cause==2

gen heart = cause==3

gen other = cause==4

gen event = (cause==cod)

***Create interactions between age group and causes***

gen agebreast = agegrp*breast

gen agecancer = agegrp*cancer

gen ageheart = agegrp*heart

gen ageother = agegrp*other

***Create dummy variables for each age cause interaction***

tab agebreast, gen(agebreast)

tab agecancer, gen(agecancer)

tab ageheart, gen(ageheart)

tab ageother, gen(ageother)

***Re-name age cause dummy variables ***

foreach var in breast cancer heart other {

rename age`var'2 age`var'1

rename age`var'3 age`var'2

rename age`var'4 age`var'3

rename age`var'5 age`var'4

}

*** Create interactions between stage and causes***

gen stagebreast = seerhistoricstage*breast

gen stagecancer = seerhistoricstage*cancer

gen stageheart = seerhistoricstage*heart

gen stageother = seerhistoricstage*other

***Create dummy variables for each stage cause interaction***

tab stagebreast, gen(stagebreast)

tab stagecancer, gen(stagecancer)

tab stageheart, gen(stageheart)

tab stageother, gen(stageother)

*** Re-name stage cause dummy variables ***

foreach var in breast cancer heart other {

rename stage`var'2 stage`var'1

rename stage`var'3 stage`var'2

rename stage`var'4 stage`var'3

}

***stset the data to tell Stata we are dealing with survival data***

stset exit, origin(dx) failure(event) scale(365.24) exit(time dx + (10*365.24))

*** Fit a flexible parametric proportional hazards model using stpm2 command***

stpm2 breast cancer heart other agebreast? agecancer? ageheart? ageother? ///

stagebreast? stagecancer? stageheart? stageother?, ///

scale(hazard) rcsbaseoff nocons ///

tvc(breast cancer heart other) initstrata(cause) ///

knotstvc(breast 1.37 2.62 4.70 ///

cancer 1.00 2.95 5.87 ///

heart 1.79 3.87 6.37 ///

other 1.95 3.95 6.46) ///

bknotstvc(breast 0.038 9.96 ///

cancer 0.04 9.96 ///

heart 0.04 9.96 ///

other 0.04 9.96)

***Predict the cumulative incidence functions, the cause-specific hazard rates, the contribution to the total mortality and the contribution to the overall hazard for each covariate pattern using stpm2cif command***

forvalues j = 1/4 {

forvalues l = 1/3 {

if `j'! = 1 {

if `l'==1 {

stpm2cif breast`j'`l' cancer`j'`l' heart`j'`l' other`j'`l', ///

cause1(breast 1 agebreast`j' 1) ///

cause2(cancer 1 agecancer`j' 1) ///

cause3(heart 1 ageheart`j' 1) ///

cause4(other 1 ageother`j' 1) haz conthaz contmort

}

if `l'! = 1 {

stpm2cif breast`j'`l' cancer`j'`l' heart`j'`l' other`j'`l', ///

cause1(breast 1 agebreast`j' 1 stagebreast`l' 1) ///

cause2(cancer 1 agecancer`j' 1 stagecancer`l' 1) ///

cause3(heart 1 ageheart`j' 1 stageheart`l' 1) ///

cause4(other 1 ageother`j' 1 stageother`l' 1) haz conthaz contmort

}

}

if `j'==1 {

if `l'==1 {

stpm2cif breast`j'`l' cancer`j'`l' heart`j'`l' other`j'`l', ///

cause1(breast 1) ///

cause2(cancer 1) ///

cause3(heart 1) ///

cause4(other 1) haz conthaz contmort

}

if `l'! = 1 {

stpm2cif breast`j'`l' cancer`j'`l' heart`j'`l' other`j'`l', ///

cause1(breast 1 stagebreast`l' 1) ///

cause2(cancer 1 stagecancer`l' 1) ///

cause3(heart 1 stageheart`l' 1) ///

cause4(other 1 stageother`l' 1) haz conthaz contmort

}

}

}

}

## Competing interests

The authors declare that they have no competing interests.

## Authors’ contributions

SRH and PCL conceived the project. SRH carried out the analysis and extended the software to enable use of the method. Both authors participated in the interpretation of the results. SRH drafted the paper, which was later revised by both authors. Both authors read and approved the final manuscript.

## Pre-publication history

The pre-publication history for this paper can be accessed here:

http://www.biomedcentral.com/1471-2288/13/13/prepub
